# One Health Evaluation of Antimicrobial Use and Resistance Surveillance: A Novel Tool for Evaluating Integrated, One Health Antimicrobial Resistance and Antimicrobial Use Surveillance Programs

**DOI:** 10.3389/fpubh.2021.693703

**Published:** 2021-08-04

**Authors:** Margaret Haworth-Brockman, Lynora M. Saxinger, Misha Miazga-Rodriguez, Aleksandra Wierzbowski, Simon J. G. Otto

**Affiliations:** ^1^National Collaborating Centre for Infectious Diseases, Winnipeg, MB, Canada; ^2^Department of Community Health Sciences, University of Manitoba, Winnipeg, MB, Canada; ^3^Faculty of Medicine and Dentistry, University of Alberta, Edmonton, AB, Canada; ^4^Antimicrobial Resistance One Health Consortium, Edmonton, AB, Canada; ^5^Human-Environment-Animal Transdisciplinary AMR Research Group, School of Public Health, University of Alberta, Edmonton, AB, Canada; ^6^Thematic Area Lead, Healthy Environments, Centre for Healthy Communities, School of Public Health, University of Alberta, Edmonton, AB, Canada

**Keywords:** program evaluation, antimicrobial resistance, surveillance, evaluation tool, complex systems

## Abstract

We describe the development, application and utility of our novel, One Health Evaluation of Antimicrobial Use and Resistance Surveillance (OHE-AMURS) tool that we created to evaluate progress toward integrated, One Health surveillance of antimicrobial resistance (AMR) and antimicrobial use (AMU) as a complex system in Canada. We conducted a qualitative inquiry into the current state of policy and programs for integrated AMR/AMU surveillance using explicit and tacit knowledge. To assess the “messy” state of public health surveillance program development, we synthesized recommendations from previous reports by the National Collaborating Centre for Infectious Diseases and the Canadian Council of Chief Veterinary Officers; conducted an environmental scan to find all federal, provincial, and territorial AMR/AMU surveillance programs in Canada; and conducted semi-structured interviews with Canadian subject matter experts. To integrate evidence from these different sources we adapted two published tools to create a new evaluation matrix, deriving 36 components of the ideal integrated AMR/AMU surveillance system. Our two-way matrix tool allowed us to examine seven common, foundational elements of sustainable programs for each component, and assign a stage of development/sustainability ranking for each component according to the matrix definitions. Our adaptable novel tool allowed for granular and repeatable assessment of the many components of a complex surveillance system. The assessment proved robust and exacting to ensure transparency in our methods and results. The matrix allows flexible assignment of program components based on program principles, and stages can be adapted to evaluate any aspect of an AMR/AMU surveillance or other multi-faceted, multi-jurisdictional system. Future refinement should include an assessment of the scope of surveillance components.

## Introduction

Monitoring progress in complex systems may require equally complex methods to capture and analyze numerous aspects of whether and how progress is achieved (1, 2). McGill et al., for example, conducted a review of methods and found 74 unique studies that described evaluations of complex systems. The authors concluded that “a range of complex systems methods can be utilized, adapted, or combined to produce different types of evaluative evidence” (3). They went on to note that methodological innovation in systems evaluation may be of particular value in generating “stronger evidence” (3).

One such complex system includes policies, practices, and resources for appropriate surveillance of antimicrobial use (AMU) and antimicrobial resistant organisms [referred to overall as antimicrobial resistance (AMR)] in human, animal, food animal production, and aquaculture. One Health, or the inseparability of the health of humans, animals, and their environment, is an important concept for surveillance that adds to this complexity (4). Assessing Canada's progress to address gaps in integrated, One Health surveillance on AMR and AMU requires interrogation of surveillance systems' objectives and data, analysis of the scope and limitations of such surveillance, and knowledge of policy developments and impediments.

We conducted a review of AMR/AMU surveillance in Canada to understand how well it has developed since reviews in 2014 in the human health realm (5) and in 2016 on animal health (6). The results of our analysis were compiled in a comprehensive report (7) and have been peer-reviewed (8). Integrated AMR/AMU surveillance across humans, animals, and the environment is highly complex and “messy” (9) because surveillance in Canada is done at a variety of sites, under different jurisdictions, with various criteria, for different purposes, and at different levels of sophistication or development. There are at least nine separate surveillance programs in Canada (not including research programs). Some, like the Canadian Integrated Program for Antimicrobial Resistance Surveillance (CIPARS) surveille aspects of human and animal health and food (10), and others, such as the Canadian Nosocomial Infection Surveillance Program (11), Canadian Fed-cattle (beef feedlot) Antimicrobial Surveillance Program (CanFASP) (12) and the Canadian Dairy Network for Antimicrobial Stewardship and Resistance (CaDNetASR) (13) are specific to only certain sectors.

In this paper, we describe our mixed methods approach to collect data on the current state of integrated One Health AMR/AMU surveillance in Canada and the process we used to find and adapt existing frameworks to analyze data gathered from documents, key informants and expert opinions effectively. In particular, we describe a novel tool we developed to assess the information gathered, by considering program scope, resource investment, and tangible progress made in the past seven years. Our purpose is to authentically report (14)—that is, to describe the narrative of our experience—on the iterative nature of the method development to suit the complex systems and allow for objective assessment (15) of AMR/AMU surveillance in Canada.

### Context

Antimicrobial resistance is a pressing global threat (16). In Canada, infection and colonization rates of pathogens such as methicillin-resistant *Staphylococcus aureus* in communities, vancomycin-resistant enterococci in hospitals, and carbapenemase-producing organisms in both settings are increasing (17). Surveillance data for AMR in animal pathogens are mostly non-existent in Canada (8). Antimicrobial resistance surveillance in Canada is considered patchy, particularly compared with the quality of surveillance done in some European countries, and is likely insufficient to deal with the enormity of the threat AMR poses in the near future (18). According to the World Health Organization, integrated, One Health surveillance of AMR/AMU must underpin efforts to protect human, animal, and environmental health (19).

In 2014, the National Collaborating Centre for Infectious Diseases (NCCID) published a report assessing the state of integrated AMR/AMU surveillance in Canada, along with 10 recommendations to address gaps and improve stewardship, surveillance and related policy in Canada (5). Two years later, the Canadian Council of Chief Veterinary Officers (CCVO), published a report evaluating current and alternative models of AMU surveillance for veterinary antimicrobials in the areas of federal, provincial, and territorial (F/P/T) antimicrobial distribution and sales data, veterinary antimicrobial purchase, sales and prescription data, and animal owner antimicrobial purchase and administration data (6). Following a national meeting on AMR surveillance in January 2019 (18), we determined to do a complete review of the current state of surveillance in Canada and to assess progress made since the recommendations made in 2014 and 2016. Our objectives were to: (1) catalog national and provincial AMR/AMU surveillance programs currently operating in Canada and; (2) assess the current state of surveillance to evaluate what progress has been made to address the gaps identified in the earlier reports (5, 6). During this work, it became clear that a reproducible evaluation matrix was needed to transparently evaluate programs in an informative way. Here, we describe our novel tool, One Health Evaluation of Antimicrobial Use and Resistance Surveillance (OHE-AMURS), for evaluating integrated, One Health AMR/AMU surveillance in Canada.

## Methods

Our project was a qualitative mixed-methods inquiry into the current state of policy and programs, drawing on explicit (codified, written evidence) and tacit (experiential) knowledge (20). The challenge we faced was to find and adapt research methods and tools to achieve both a program assessment and analysis of policies in combination for a complex One Health surveillance system.

We used three data collection methods, described in detail in the final report to the NCCID (18). Briefly, we first synthesized and updated the recommendations from the previous reports. We then conducted an environmental scan to find all federal, provincial and territorial AMR/AMU surveillance programs in Canada. Thirdly, we conducted semi-structured interviews with Canadian subject matter experts.

### Developing a Novel Tool for Analysis

Analysis began with synthesizing and refining the original recommendations from the 2014 report (5) to align with the current surveillance landscape in Canada. Incorporating findings from the 2016 report (6) and information from the environmental scan and interviews, we refined eight overarching requirements for integrated AMR/AMU surveillance. Within each of the requirements, we identified components that define the structures necessary to fulfill the requirement as a whole, based on the existing surveillance programs. While two requirements had one component each, others had five or more, adding up to 36 components in all needed for comprehensive, sustained surveillance of AMR and AMU in humans and animals (Table 1).

**Table 1:** Program Requirements and their Components for Integrated, One Health AMR/AMU surveillance in Canada.

**Requirements for integrated surveillance (8)**	**Components within requirements (36)**
1. National integrated AMR/AMU surveillance system	Federally coordinated, cross-sectoral, integrated system of AMR/AMU surveillance
	Standardized surveillance definitions, metrics, and performance indicators across provinces, territories, and federally
	Support for integrated provincial and territorial initiatives
2. Maintain and increase resources for existing AMR/AMU surveillance programs	Resources/funding: multi-sector plan for comprehensive surveillance
3. National AMR data warehousing initiative	AMR data warehouse (AMR NET; based on the EU model)
4. National human AMR and AMU surveillance	AMR surveillance (Human nosocomial pathogens CNISP; foodborne pathogens in humans CIPARS)
	AMR surveillance for other human pathogens (e.g., pathogens not covered by CNISP/CIPARS, community-acquired pathogens)
	Centralized collation of hospital AMU data (CNISP is the only AMU program evaluated)
	Human antimicrobial distribution And prescribing data (IQVIA data)
	Non-CNISP point prevalence surveys of AMR and AMU in hospitals (CNAPP, academia, pharmaceutical, and WHO projects)
5. National animal AMR and AMU surveillance	Collaborative national working group on animal AMR/AMU surveillance
	CIPARS—antimicrobial sales/distribution data for animals
	CIPARS Farm-level AMR/AMU surveillance—swine, broilers, chickens and turkeys
	CIPARS Farm-level AMR/AMU surveillance—feedlot cattle (Canadian fed-cattle (feedlot cattle) antimicrobial surveillance program—CanFASP).
	Canadian dairy network for antimicrobial stewardship and resistance—farm-level AMR/AMU data
	Farm-level AMR/AMU surveillance—cow-calf
	Veterinary or farm-level AMR/AMU surveillance for remaining food and companion animals (small animals, equine)
	Department of fisheries and oceans collection of AMU data from aquaculture producers in Canada
	CIPARS animal clinical, abattoir and retail AMR components
	AMR Surveillance of veterinary pathogens
	Reporting requirements for antimicrobial susceptibility data from vet labs (AMR Net)
	AMR Surveillance in soil and water
	CIPARS Crop AMU surveillance
	CIPARS Aquaculture AMU surveillance
6. Collection of antimicrobial use indication data	Swine/broiler chicken/turkey on-farm programs provide indication data (CIPARS)
	Beef feedlot indication data (CIPARS)
	Canadian dairy network for antimicrobial stewardship and resistance (CaDNetASR)
	Veterinary prescribing surveillance (CVMA project)
	Human antimicrobial indication data (primarily CARSS IQVIA data: other sources under consideration)
7. Timely and integrated national reporting of AMR/AMU data	CARSS—human and animal AMR/AMU report
	CIPARS—human and animal AMR/AMU report
	CIPARS Interactive display dashboard for human and animal AMR/AMU reporting
8. Formal recognition of one health policy for antimicrobial stewardship	Policy to recognize “One Health” as a priority for Canada
	Legislated requirement for animal antimicrobial sales reporting by all manufacturers, importers and compounders of 2019
	Elimination of the “Own Use Importation” provision for medically important antimicrobials
	Elimination of non-approved “active pharmaceutical ingredient” use and importation of medically important antimicrobials

*AMR, antimicrobial resistance; AMU, antimicrobial use; CNISP, Canadian Nosocomial Infection Surveillance Program; CIPARS, Canadian Integrated Program for AMR Surveillance*.

Faced with an abundance of information in very different forms and a recognized lack of available, detailed methods and tools to evaluate surveillance programs (21), we searched for an analysis method or tool to enable us to describe the stages of program development for the requirement components systematically, and then to assess surveillance program sustainability with the granularity we required. At the time of this analysis (2019), we did not find any tools that met our specific needs for comprehensive evaluation of all integrated, One Health elements of an AMR/AMU surveillance program. In March 2021, Aenishaenslin et al. published a new Integrated Surveillance System Evaluation (ISSE) framework, which is a conceptual framework for evaluating the integration of One Health surveillance systems for AMR and AMU (22). Very useful for future evaluations, it presented a framework for developing an evaluation protocol but did not provide an explicit tool in itself, thus would not have suited our immediate purpose. In iterative searches of program evaluation literature, we found, adapted and trialed a situation analysis tool for action on AMR, developed by the World Health Organization (WHO) South East Asia Regional Office (SEARO) which addressed progress, that is a “stepwise, incremental approach of a SEARO country toward implementing the WHO Global Action Plan on AMR” (23). Parathon et al. report implementing the tool to assess progress on Indonesia's national AMR prevention and containment, using progress phases categorized as exploration and adoption; programme installation; initial implementation; full operation; and sustainable operation. We adapted the tool to assess the relative stages of program development for integrated AMR/AMU surveillance in Canada, with the slightly refined program development headings of exploration, program adoption, initial implementation, full operation, and sustainable operation.

However, in application, we found that integrated AMR/AMU surveillance programs could have significantly different stages of development across various program aspects so a way to separate discrete program aspects for individualized assessment was required. We identified a public health framework developed by Schell et al. to assess the “sustainability capacity” of public health program components (24). Schell et al. defined sustainability capacity as, “the existence of structures and processes that allow a program to leverage resources to effectively implement and *maintain* evidence-based policies and activities” (emphasis added). The authors' original nine domains were: political support, funding stability, partnerships, organizational capacity, program evaluation, program adaptation, communications, public health impacts, and strategic planning. We determined we did not have the necessary data or frame of reference to evaluate political support for the components under the eight surveillance program requirements, so substituted evaluation of enabling policy. We also decided not to evaluate public health impacts of AMR/AMU surveillance, in part because of a recent, parallel work assessing the socioeconomic, health systems and health outcome costs of AMR published by the Council of Canadian Academies (25). Thirdly, we did not include program evaluation as there was little information available about this element for national AMR/AMU surveillance. Thus, our modification arrived at seven program elements for our evaluation: funding, organizational capacity, partnerships, program adaptability, communication, strategic planning and enabling policy (Table 2).

**Table 2:** Comparison of our One Health Evaluation of Antimicrobial Resistance Surveillance (OHE-AMRU) tool to existing methods that were adapted to fit our purpose.

**Original situation analysis tool**	**Novel tool adaptations**
**Program development phases [Parathon et al.**, **(****23****)]**	
•Exploration and adoption, •Programme installation, •Initial implementation, •Full operation, •Sustainable operation	•Exploration, •Program adoption, •Initial implementation, •Full operation, •Sustainable operation
**Sustainability capacity [Schell et al.**, **(****24****)]**	
•Political support, •Funding stability, •Partnerships, •Organizational capacity, program evaluation, •Program adaptation, communications, •Public health impacts, •Strategic planning	•Funding, •Organizational capacity, partnerships, •Program adaptability, communication, •Strategic planning and •Enabling policy.

**Figure 1:**
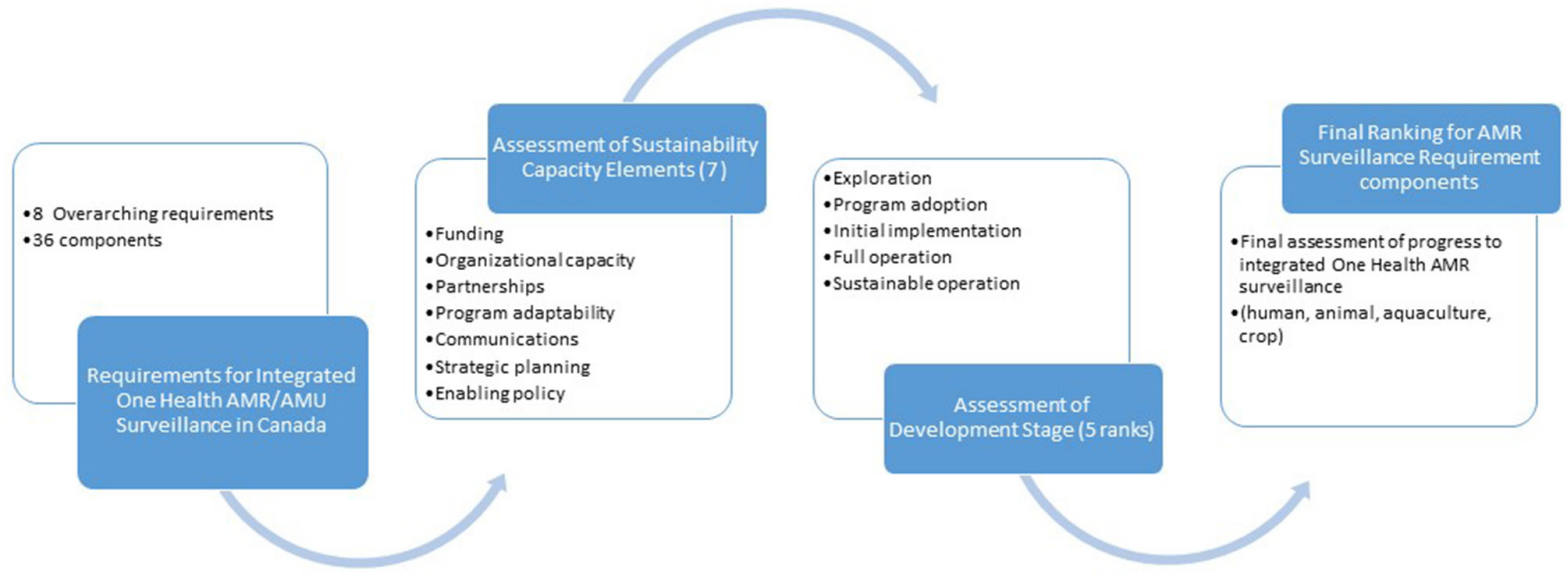
Step-wise process used to develop and use the OHE-AMURS novel tool.

We combined the five elements of program sustainability with the seven stages of program development (Figure 1) into a final OHE-AMURS matrix with and developed definitions for each (Table 3). This novel matrix allowed us to assess progress made toward the eight requirements for national, integrated AMR/AMU surveillance in Canada.

**Table 3:** The matrix for the One Health Evaluation—Antimicrobial Use and Resistance Surveillance (OHE-AMURS) tool to evaluate national, integrated antimicrobial resistance and antimicrobial use surveillance program component elements in Canada.

**Common program sustainability elements**	**Stage of program development rankings**			
	**1-Exploration**	**2-Program adoption**	**3-Initial implementation**	**4-Full operation**	**5-Sustainable operation**
Funding	No or limited funding for pilot program	Initial funding for pilot, but funding for broader program planning and operation unavailable	Time-limited, short-term, dedicated funding for program planning and operation within a defined interval	Time-limited, longer-term dedicated funding allows for program planning and operation within defined interval	Permanent, dedicated funding that enables long-term program planning (not time-limited)
Organizational capacity	Limited or no dedicated resources to launch program. Full program planning and operation not possible	Time-limited, dedicated resources to launch program, but capacity for full program planning and operation not available	Time-limited, short-term, dedicated resources to effectively manage the program within a defined period	Time-limited, long-term, dedicated resources to effectively manage the program within defined interval	Permanent, dedicated resources to effectively manage program over long-term
Partnerships	Formal and informal connections with key stakeholders starting	Time-limited, formal or informal connections between the program and key stakeholders in development	Short-term, time-limited, formal/informal connections in place or in development	Long-term but time-limited formal and informal connections	Long-term, formal connections in place
Program adaptability	No ability for improvement, expansion or response to emerging threats	Limited ability for improvement and expansion; no ability to respond to emerging threats	Limited ability for program improvement, expansion, and response to emerging threats	Program can moderately improve, expand, and respond to emerging threats, given available funding and resources	Program can improve, expand, and respond to emerging threats
Communications	Limited, informal communication to stakeholders, decision-makers and the public	Limited process for disseminating pilot outcomes and activities to few audiences	Process for periodic dissemination of program outcomes and activities in development	Periodic dissemination of program outcomes and activities in place	Strategic and timely dissemination of program outcomes and activities with stakeholders, decision-makers and public
Strategic planning	Program direction, goals, and implementation strategies developing	Program direction, goals and strategies in place for program implementation	Program direction, goals, and strategies are implemented, but no plan for ongoing review and updating	Program direction, goals and strategies in place for funding period and resources; process for review in place or developing	Program direction, goals and strategies in place and subject to regular review
Enabling policy	No policy, or in early stages of discussion	Policy in development but not implemented; may or may not have stakeholder input	Policy exists or in early implementation; data sharing, standardization between F/P/T levels and stakeholders limited or non-existent	Policy in place for limited data sharing and standardization among F/P/T levels (including some stakeholders)	Policy allows for effective and efficient data sharing and standardization among F/P/T; respects and includes all relevant stakeholders

The common program elements for evaluation are in the left column and the rankings for stages of program development are in the top row. Criteria for every element-stage combination rank are defined. Criteria are adapted from Parathon et al. and rankings are adapted from Schell et al. F/P/T, federal/provincial/territorial.

We defined the five stages of development for each of the seven sustainability capacity elements through iterative discussions by the investigation team. We created these definitions in advance of our assessment of program components to ensure objectivity based on the data and information collected.

We assigned one of the five stages of program development to every element of a surveillance program component, based on our analysis of available literature, program reports, and information from the interviews. The investigation team made these assignments through iterative discussion, with justifications provided in a compiled table of results (8). Where we were uncertain, we had follow-up conversations with key experts who were knowledgeable about specific programs. In some cases, where our initial definitions did not allow for clear differentiation between development stages, we refined our definitions and revised any initial assessments to program elements.

Our process and the development of our new assessment tool allowed us to populate our resulting table with additional information to allow replication and interpretation.

### Validation by Experts

We compiled a draft summary of our results and distributed it to key interview respondents for their review and validation between December 2019 and January 2020. The reviewers included representatives from the largest national human and animal surveillance programs, co-chairs of national AMR/AMU surveillance task forces, and the authors of the 2014 and 2016 reports. We asked our reviewers to comment on (a) whether our list of surveillance programs was complete; (b) the accuracy of our descriptions of the necessary surveillance program components under each of the requirements; and (c) the completeness and accuracy of our detailed assessments for every development stage and element of the components.

Experts' comments and recommendations were used to amend assignments and rationale for each program assessment component. In some cases, reviewers' rankings of development stage and our justifications were substantively different from those of our investigation team. We followed up with these experts by email and telephone with reviewers and key subject matter experts (February-March 2020) to make adjustments to our findings as applicable.

### Final Products and the Value of the Novel Tool

We completed our final analysis of our findings across the eight requirements for integrated, One Health AMR/AMU surveillance in Canada in the summer of 2020 and incorporated additional developments up to October 12, 2020. The importance of developing this novel tool was underscored by our results, as we found that many programs had elements assessed at differing stages of development toward the various recommendations. As well as producing a comprehensive spreadsheet with all rankings and the rationale for those rankings, we created a simplified, color-coded “heat map” that allows for rapid interpretation of the overall state of AMR/AMU surveillance and facilitates comparison across the domains of the tool (7, 8). These products should allow for timely updates and replication of our evaluation.

## Discussion and Conclusion

Our objective was to find a suitable method to assess the messy and complex nature of human, animal, crop and aquaculture AMR and AMU surveillance in Canada accurately and adequately, and in a way that could be understood and repeated by others. Other authors have described the challenges of finding tailored methods to evaluate processes and outcomes of complex and messy systems that are based on several sources of data and which must be contextualized (1, 26).

A systematic review of evaluation approaches for surveillance systems highlighted that there are a number for public and animal health (21). The review categorized these approaches as frameworks, guidelines, methods, and tools based on the level of instructional guidance that they provided to the user for implementation. The authors suggested that methods (what components/elements to assess and how to assess them) and tools (practical elements to conduct the assessment) are required for full utilization. However, of the 15 approaches reviewed, only three included such methods and tools for implementation, highlighting the need for practical tool development in this field. None of the reviewed approaches considered the complexity of One Health AMR and AMU surveillance, which includes components from humans, animals, and the environment.

Publications from the international CoEval-AMR Network (27), released in March 2021, parallel the processes we undertook to test and refine evaluation frameworks and tools for AMR/AMU surveillance. Aenishaenslin et al., for example, reported on their development of the ISSE framework and piloted the tool to review one Canadian AMR surveillance program, CIPARS, a sentinel surveillance program for animal and human health (22). The authors began their process with CIPARS program experts to determine an evaluation framework and then conducted the program evaluation in a stepwise fashion. Results included a determination of actions and policy changes arising from the evaluation. However, this framework would not have provided the explicit tool we required to evaluate specific surveillance recommendations and components. Our method and tool did not attempt to comment on actions or next steps for the specific AMR/AMU programs, as our focus was on the overall development and sustainability of programs to achieve effective integrated monitoring. The utility and feasibility of 12 other tools for surveillance or One Health evaluation were assessed by the CoEval-AMR Network project team since 2019 (28). These included AMR/AMU surveillance tools previously reviewed by Nielsen et al. (29) using 10 criteria to rank the utility and feasibility of the frameworks. It would be valuable to use criteria such as Nielsen's to compare the utility of our complex systems assessment tool with other evaluation tools, depending on what is being evaluated. None of these tools were comprehensive that covered all the One Health elements of an integrated AMR/AMU surveillance programs, nor did they allow for the customization of requirements and components that our tool provided.

Our novel OHE-AMURS tool adapted two other systems evaluations to include the elements for assessment with definitions of the sustainability stages. The tool allowed us to assess every component of the eight recommendations for integrated AMR/AMU surveillance with the granularity needed. There was, for example, clear utility in defining a temporal rank of “stage of development,” which also provided a built-in “road map” for what progress would look like and illuminates ultimate goals. The matrix is complex but it allowed us to conduct a nuanced assessment that was robust for iterative review. We found it was ultimately an exacting tool and a way to engage with stakeholders while ensuring transparency in our methods and in the results.

Our tool is also flexible and adaptable and can be used for repeated evaluation of the future state of AMR/AMU surveillance in Canada and other multi-jurisdiction regions. The two-way element-stage of development classification and definitions and lack of pre-determined underlying components provides utility for this tool to be applied to evaluation of other integrated, One Health AMR/AMU surveillance programs. This flexibility was evident when applied to the 36 components and eight requirements across the One Health sectors, which represents the epitome of a “messy” system (9). We did, however, require an iterative process to develop both the matrix definitions and the final assessment of components. As we worked through the evaluation, we realized that some definitions required clarification to allow for adequate discrimination between the stages of development. The review from subject matter experts allowed us to refine our assessments of development for every component. This increased our confidence that we had assessed all of the required components of the integrated AMR/AMU surveillance system with the best available information at the time.

One potential weakness is that in a few instances our evaluation criteria ranked program components highly, but in fact the components were not comprehensive by our own assessment. It became clear that our evaluation tool was missing a means to assess the scope and comprehensiveness of program elements. Some elements ranked highly when using the stage of development definitions, but ultimately were too limited in scope to be considered truly comprehensive and integrated for national AMR/AMU surveillance for Canada. We recommend that for future applications, every program element be evaluated for scope and comprehensiveness using a ranking system such as: Sufficient, Partial, or Insufficient. The criteria should be defined as part of the evolution of this novel tool for future AMR/AMU surveillance program evaluations.

Our novel OHE-AMURS tool was well-suited to provide an assessment of the current status of integrated, One Health AMR/AMU surveillance programs and can be used both for future evaluations at all levels, and for other complex systems with multiple elements that may be at different stages of development. Its flexibility and iterative nature allowed for a comprehensive evaluation of the current state in Canada. With modifications to incorporate assessments of scope and comprehensiveness, OHE-AMURS will serve as a useful tool for future reviews of the AMR/AMU surveillance infrastructure in Canada. Regular evaluations of complex national AMR/AMU surveillance programs are essential for continued momentum to improve monitoring, provide data for action, and mitigate antimicrobial resistance in Canada.

## Data Availability Statement

The original contributions presented in the study are included in the article/supplementary material, further inquiries can be directed to the corresponding author/s.

## Author Contributions

MH-B and SO conceived of the paper, wrote the drafts, and made final revisions. LS, AW, and MM-R contributed content and reviewed all drafts. All authors contributed to the article and approved the submitted version.

## Author Disclaimer

The views expressed herein do not necessarily represent the views of the Agency.

## Conflict of Interest

The authors declare that the research was conducted in the absence of any commercial or financial relationships that could be construed as a potential conflict of interest.

## Publisher's Note

All claims expressed in this article are solely those of the authors and do not necessarily represent those of their affiliated organizations, or those of the publisher, the editors and the reviewers. Any product that may be evaluated in this article, or claim that may be made by its manufacturer, is not guaranteed or endorsed by the publisher.
